#  Development of an RP-HPLC-UV Method for Simultaneous Detection of Nimodipine and its Metabolite in Cerebrospinal Fluid of Rat 

**Published:** 2017

**Authors:** Farzaneh Sotoudegan, Mohsen Amini, Mehrdad Faizi, Reza Aboofazeli

**Affiliations:** a *Department of Pharmaceutics, School of Pharmacy & Protein Technology Research Center, Shahid Beheshti University of Medical Sciences, Tehran, Iran. *; b *Department of Pharmaceutical Chemistry, Faculty of Pharmacy, Tehran University of Medical Sciences, Tehran, Iran. *; c *Department of Pharmacology and Toxicology, School of Pharmacy, Shahid Beheshti University of Medical Sciences, Tehran, Iran.*

**Keywords:** Nimodipine, Nimodipine oxide, HPLC, Cerebrospinal fluid, Polymeric micelle

## Abstract

A rapid, simple and reproducible HPLC method was developed and validated for the analysis of nimodipine (NM) and/or its metabolite, oxidized nimodipine (OX–NM) in rat cerebrospinal fluid (CSF) and artificial CSF. The NM and OX–NM were eluted in less than 10 min with no interferences from the endogenous CSF peaks. Analysis was carried out on a Eurospher Performance (RP-C18, 250 × 4.6 mm) column and UV detection at 236 nm. The mobile phase consisted of acetonitrile and water (70:30 v/v, respectively) with a flow rate of 1 mL/min. Limit of detection was 0.1 μg/mL for OX-NM. The calibration curve was linear over the concentration range of 0.5-10 µg/mL and analytical recovery was more than 95%. The coefficients of variation for intra-day and inter-day assays were less than 5%.

## Introduction

Nimodipine (NM) is a dihydropyridine calcium channel blocker which selectively regulate calcium channels, dilate cerebral arterioles and thereby increase cerebral blood flow (1) and is indicated for the treatment of a range of cerebrovascular disorders (2, 3). However, its clinical performance is limited, because it is subjected to extensive first-pass metabolism in liver which leads to a very low oral bioavailability and hence only a small fraction of the administered dose is delivered to the brain (4). Mean cerebrospinal fluid (CSF) levels have been reported to be amounted 0.3 ± 0.2 µg/L in patients with subarachnoid hemorrhage whose mean plasma concentrations were 76.9 ± 34.0 µg/mL (5). Also, NM is highly lipophilic which necessitates its administration as a parenteral formulation containing organic solvents, such as ethanol. NM ethanolic injections may cause local adverse reactions (including pain, inflammation, phlebitis) at the administration site (6). Therefore, looking for alternative routes of administration to improve the therapeutic effects seems strongly helpful in case of brain medication. 

One of the novel strategies is to pass drug molecules through BBB by means of encapsulation in polymeric micelles. Basically, polymeric micelles are kinetically stable nanoparticles with a core-shell structure which are made from amphiphilic block copolymers with hydrophilic and hydrophobic chains that self-assemble in water above the critical micelle concentration. The hydrophobic core of these nano-carriers could serve as a microenvironment for solubilizing poorly water soluble drugs and protect the enclosed compounds from inactivation in biological media, while exposing their hydrophilic shells to the external environment. It has been reported that these systems could enhance the drug diffusion across blood brain barrier (7, 8). 

**Figure 1:**
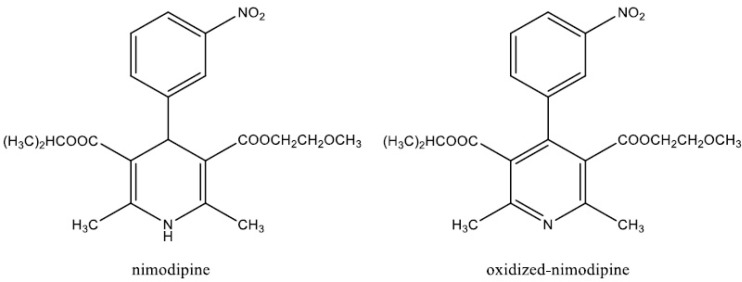
Chemical structure of nimodipine and oxidized-nimodipine

As a part of our main research to develop a delivery system for NM, based on its entrapment by the polymeric micelles, we needed to develop an analytical method for the determination of NM and its metabolite, oxidized NM (Figure 1) in CSF. Among the different methods reported in the literature for the analysis of NM in body fluids, high-performance liquid chromatographic (HPLC) methods were shown to be more efficient. Few methods which utilized gas chromatography (GC) with electron-capture (9) and nitrogen detection (10), were complicated by uncontrolled oxidation of NM at high temperatures. A highly sensitive and selective HPLC method for the determination of NM and its major metabolites has been described in the literature (9), however, it requires a large amount of plasma and an elaborate and lengthy sample preparation method. In the present study, a simple and rapid HPLC assay for the determination of NM and its metabolite (OX–NM) in rat CSF was described. We showed the potential use of the developed technique for quantitatively monitoring NM and OX–NM in rat CSF after injection of drug-loaded polymeric micelle formulations.

## Experimental

*Materials*

NM was purchased from Sigma-Aldrich (St. Louis, MO, USA). HPLC-grade acetonitrile was obtained from Merck Chemical Company (Darmstadt, Germany). Water was prepared by a Millipore Milli-Q Plus Water Purification System. All other reagents used were of analytical grade. Hydrophilic Pluronic^®^ block copolymers (PBC), including P85 and F127 were commercially available from BASF 

(New Jersey. NJ, USA).

*Preparation of NM-loaded polymeric micelles*

Pluronic^® ^P85 (0.6 g), Pluronic^® ^F127 (0.4 g) and 2 mg NM were added simultaneously to the 10 mL deionized water and the solution was stirred at 100 rpm for 12 h at 25 °C. The precipitated NM was separated from the polymeric micelles solution by filtering through the 0.45 µM filter membranes (NYLON Membrane Filter, Vertical).

*Chromatographic system and conditions*

A Waters HPLC System (Waters Corporation, MA, USA), equipped with a Waters 600E Multi-solvent Delivery System, a Rheodyne injector and a Waters 486 Tunable Absorbance Detector, was used for the analysis. The separation was performed, using an Eurospher column (RP-C_18_, 250 × 4.6 mm, Knauer, Germany). An isocratic elution system was employed with a mobile phase consisted of acetonitrile and water (70:30 v/v) which was freshly prepared and degassed by ultra-sonication. The flow rate was set at 1 mL/min^−1^. The mobile phase was not allowed to recirculate during the analysis. Chromatograms were monitored by UV detection in the range of 200-400 nm and displayed at a single wavelength of 236 nm. 

*Preparation of artificial CSF*

To prepare the artificial CSF (osmolarity of 320 mOsm, pH 7.4), 150 mM of NaCl, 3.5 mM of KCl, 2 mM of MgCl_2_, 1.2 mM of CaCl_2_, 10 mM of HEPES and 20 mM of glucose were added to 200 mL distilled water. The solution was stirred for 120 min at room temperature (11).

*Synthesis of NM oxide (OX-NM)*

NM solutions were stored and analyzed in complete darkness. For the synthesis of OX–NM, 4 mg of MnO_2_ was added to 20 mL dichloromethane containing 1 mg of NM and the mixture was stirred overnight. The obtained solution was filtered and then purified by a column chromatographic method. The ^1^H and ^13^C NMR techniques were used to confirm the aromatization of the dihydropyridine (DHP) ring. 

*Standard solutions*

Standard solutions were prepared at concentrations of 0.5, 1, 2, 5 and 10 µg mL^−1 ^of OX–NM by serial dilution with the artificial CSF. 

*Method validation*

The developed method was validated in terms of selectivity, linearity, accuracy and precision (intra and inter-day variability), limits of detection (LOD) and quantification (LOQ) and recovery according to the accepted guidelines (12, 13).

*a) Selectivity*

In order to verify the selectivity of the method, blank natural CSF samples aspirated from three different rats and the prepared artificial CSF were analyzed by the procedure described above and possible interferences with the analytes were checked by visual comparing of the chromatograms with those obtained from the respective samples

*b) Accuracy*

To evaluate the accuracy of the method (measured as the percent recovery of OX–NM), solutions with concentrations of 3 and 7 µg/mL in three distinct sets of media, namely acetonitrile/water mixture (70:30 v/v), artificial CSF and CSF aspirated from rats, were prepared and analyzed by the HPLC technique. This procedure was performed in triplicate. 

Furthermore, in order to reassure the consistency of the results obtained from the aspirated CSF with those from the artificial CSF, NM and OX–NM were dissolved in both media and the solutions, analyzed by the HPLC and the resulting chromatograms were inspected visually.

*c) Precision*

Within- and between-day precision of the method were determined by analyzing six replicates of samples on the same day and three different days, respectively, at three concentration levels (1, 2 and 5 μg/mL) of OX-NM in the artificial CSF and expressed as the relative standard deviations (% RSD) for each concentration level.

*d) Linearity assessment*

To evaluate the linearity of the HPLC method, calibration curves were constructed using freshly prepared spiked samples, over a concentration range of 0.5-10 µgmL^−1 ^of OX–NM in the artificial CSF, and by plotting the peak-area versus the nominal concentration of OX-NM. Linearity of the method was established by the least-squares linear regression. 

*e) Limit of quantification (LOQ) and limit of detection (LOD)*

The quantification limit of an analytical procedure was considered as the lowest amount of the analyte in a sample which can be quantitatively determined with suitable precision (% RSD) and accuracy (% recovery). The detection limit was assessed as the lowest amount of the analyte in a sample which can be detected but not necessarily quantitated as an exact value.  

*Method application*

A rat model was employed to evaluate the ability of the polymeric micelles for passing through the BBB. Adult male Wistar rats weighting between 200-250 g were supplied by Experimental Animal Laboratory of School of Pharmacy, Shahid Beheshti University of Medical Sciences (Tehran, Iran). Animals were provided with a standard laboratory diet and all experiments were carried out in accordance with guidelines evaluated and approved by the ethics committee of Shahid Beheshti University of Medical Sciences (Tehran, Iran). Drug-loaded polymeric micelles were injected intraperitoneally (IP) in 3 animals and after 40 minutes, their spinal fluids were aspirated (14) and assayed by the HPLC method described. 

## Results and Discussion

The aim of the main project was to develop and characterize a new brain delivery system, capable of passing NM through the BBB. It was hypothesized that Pluronic polymeric micelles may be able to transport NM across the BBB and enhance its brain delivery for treatment of CNS disorders. As the preliminary step, we specifically developed our laboratory polymeric micelle formulations loaded with NM and set up an HPLC method capable of monitoring the amount of NM in rat CSF. The proposed analytical method should also be able to detect OX–NM as the main and specific metabolite of NM in CNS, since it has been shown that the amount of OX–NM measured in CSF could be used as a marker for the amount of NM passed through the BBB (15). To the best of our knowledge, no analytical method has yet been reported for simultaneous determination of NM and its metabolite in the rat CSF. Thus, this study describes the development of a highly sensitive and selective RP-HPLC-UV method for the assay of NM in CSF, following the solubilization into polymeric micelles.

**Table 1:** Results of intra- and inter-day variation assessment expressed as RSD.

**OX-NM Spiked concentration (μg/mL)**	**Between-day assay (n = 6)**			**Within-day assay (n = 6)**		
	**Mean**	**SD**	**RSD (%)**	**Mean**	**SD**	**RSD (%)**
1	1.2	0.06	4.9	1.1	0.06	5.1
2	2.3	0.1	4.3	1.9	0.06	3
5	5.1	0.2	3	5.2	0.06	1.1

**Table 2:** Accuracy assessment expressed as percent of recovery (n = 3)

**Parameter**	**OX-NM Concentration**					
	**3 (μg/mL)**			**7 (μg/mL)**		
	**rat CSF**	**artificial CSF**	**acetonitrile: ** **water (70:30 v/v)**	**rat CSF**	**artificial CSF**	**acetonitrile:** **water (70:30 v/v)**
Recovery (%)	95	98	97	96	98	100

**Figure 2:**
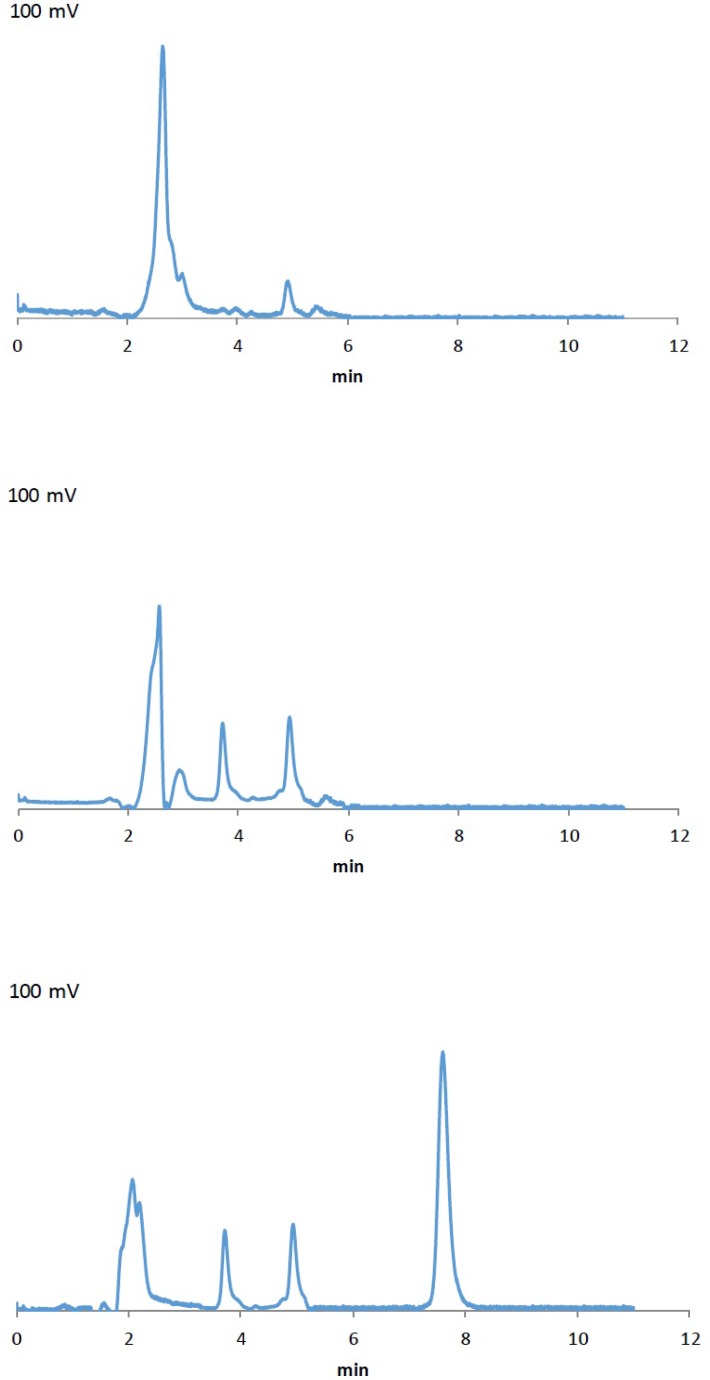
HPLC chromatograms, a) blank artificial CSF, b) blank CSF aspirated from rats, c) a sample of CSF aspirated from rats after the injection of the NM-loaded polymeric micellar solution

*Structure elucidation*

The results obtained from the NMR spectrum showed that OX–NM was synthesized successfully. ^1^HNMR, δ_1 _(ppm): (8.25, d, J = 8Hz , 1H , H4- phenyl), 8.19 (S, 1H, H2-phenyl), 7.65 (d, J =7.8 Hz , 1H, H6- phenyl) , 7.59 (d , J = 7.8 H2 , 1H , H5- phenyl), 4.90 – 4.98 (H, J = 7.0 Hz , 1H , -O-CH(CH_3_)_2_), 4.16 (δS, 2H , -O-CH_2_-CH_2_-O-CH_3_), 3.35 ( t, J = 7.5 Hz, -O-CH_2_-CH_2_-O-), 3.22 (S, 3H, OCH_3_), 2.63 (d, J =7.0 Hz , 6H, CH(CH_3_)_2_). ^13^CNMR, δ (ppm): 167.1, 166.6, 156.1, 156.0, 147.9, 143.5, 138.0, 134.5, 129.2, 126.9, 126.3, 123.6, 123.3, 69.8, 69.6, 61.3, 58.7, 23.1, 23.0, 21.29.

*Method development*

The ultraviolet spectra of OX-NM and NM depicted a maximum absorption wavelength at 236 nm. Solvent mixtures of acetonitrile/water or methanol/water with various volume ratios were examined and a mixture of acetonitrile/water at the ratio of 70:30 (v/v) was selected as the optimum mobile phase. The obtained chromatogram showed a rapid separation with the retention times of 7.5 and 6.9 min for OX-NM and NM, respectively (chromatograms are not presented). Once the consistency of the results obtained from the aspirated CSF with those obtained from the artificial CSF was confirmed, the artificial CSF was used in order to construct the calibration curve and develop the HPLC method.

*Validation of the method*

As mentioned earlier, the proposed method was fully validated according to international guidelines (12, 13). Blank polymeric micelles, drug-free artificial CSF and CSF aspirated from rats were also analyzed by this method. No interfering peak at the retention times of 6.9 and 7.5 min was observed (Figure 2a and 2b). As illustrated in Figure 2c, the peak at 7.5 min related to OX-NM was easily spotted, suggesting the specificity of the method and its suitability for the routine quality control analyses of OX-NM in rat CSF. 

Although initial attempts showed adequate resolution between the peaks relevant to NM and OX-NM, however, as seen in Figure 2c (the chromatogram of the CSF aspirated from rats after injection of drug-loaded polymeric micelles), no peak was observed at 6.0 min. This could be attributed to either a low level of NM in the aspirated CSF or the complete metabolism of NM into its metabolite OX-NM. Since this metabolite can be produced by the oxides/hydrolase enzyme which is only found in the CNS, it can be considered as an appropriate index for the determination of NM passage through the BBB (16). It should be noted that the retention times for NM and OX-NM dissolved in the aspirated CSF were consistent with those obtained for NM and OX-NM dissolved in the artificial CSF. 

A good linearity was observed over the concentration range of 0.5-10 µgmL^−1 ^of OX–NM between peak-area and the respective OX-NM concentrations in the artificial CSF. The regression equation was determined as y = 0.0409 x + 2.3513 with an excellent correlation coefficient and RSD of 1% for the slope. 

The precision was also determined by calculating RSD values of six consecutive injections of the working standard solutions. The RSD of peak area of OX-NM was less than 5% which is acceptable for the routine measurement (Table 1).

We assessed the accuracy of the method by repeated analytical recovery of known concentrations of OX-NM (3 and 7 µgmL^−1^) in artificial CSF, CSF aspirated from rats and acetonitrile/water mixture (70:30 v/v). As presented in Table 2, coefficients of variation were found to be less than 5%. The LOQ was calculated as 0.1 μgmL^−1^ for OX-NM, showing a high sensitivity for drug monitoring and pharmacokinetic studies.

## Conclusion

A rapid and sensitive HPLC method for the identification and assay of NM and its main metabolite, OX-NM, in rat CSF was developed and validated. 

The use of a simple procedure for sample preparation makes the proposed method a fast and reliable one for future pharmacokinetic studies.
